# Proteinuria and the risk of Incident atrial fibrillation according to glycemic stages: a nationwide population-based cohort study

**DOI:** 10.1186/s12933-025-02590-2

**Published:** 2025-01-24

**Authors:** Muhan Yeo, So-Ryoung Lee, Eue-Keun Choi, JungMin Choi, Kyung-Yeon Lee, Soonil Kwon, Hyo-Jeong Ahn, Bong-Seong Kim, Kyung-Do Han, Seil Oh, Gregory Y. H. Lip

**Affiliations:** 1 Department of Internal Medicine, Seoul National University College of Medicine, 101 Daehak-ro, Jongno-gu, Seoul, Republic of Korea; 2 Department of Internal Medicine, Seoul National University Hospital, Seoul, Republic of Korea; 3 Department of Internal Medicine, SMG-SNU Boramae Medical Center, Seoul, Republic of Korea; 4 Statistics and Actuarial Science, Soongsil University, 369 Sangdo-ro, Dongjak-gu, Seoul, Republic of Korea; 5 Liverpool Centre for Cardiovascular Science, University of Liverpool, Liverpool John Moores University and Liverpool Chest and Heart Hospital, Liverpool, UK; 6 Department of Clinical Medicine, Aalborg University, Aalborg, Denmark

**Keywords:** Atrial fibrillation, Diabetes Mellitus, Proteinuria

## Abstract

**Background:**

Diabetes mellitus (DM) and proteinuria each independently raise the risk of atrial fibrillation (AF). We aimed to investigate the relationship between proteinuria and the risk of incident AF across glycemic stages.

**Methods:**

A cohort of 4,044,524 individuals without prior AF and type 1 DM was selected from the 2009 Korean National Health Insurance Service health checkup data. The individuals were categorized into five glycemic stages: normal, prediabetes, new-onset DM, early DM (< 5 years), and late DM (≥ 5 years). Proteinuria was graded using a urine dipstick test. The development of incident AF was tracked until 2023.

**Results:**

Overall, the cohort (mean age 47 ± 14 years, 44.8% female) showed increasing annual AF incidence rates from 2.05 to 7.22 per 1000 person-years from normal to late DM (*p* < 0.001). Incidence rates increased from 2.46 to 8.18 per 1000 person-years with increasing proteinuria (*p* < 0.001). Adjusted Cox regression models revealed a heightened AF risk with higher proteinuria across all glycemic stages (adjusted hazard ratios for urine dipstick 3+/4+: 1.58, 1.64, 2.39, 2.12, and 2.53 for normal, prediabetes, new-onset DM, early DM, and late DM, respectively). The proteinuria-AF association was more pronounced in individuals with DM than in those without DM but was similar among the new-onset and established DM groups.

**Conclusions:**

Proteinuria is an independent and significant risk factor for incident AF at all glycemic stages. The risk of incident AF in patients with DM can be stratified by measuring the level of proteinuria rather than comparing the duration of DM. Tailoring clinical strategies to proteinuria level could potentially mitigate this risk, improving patient outcomes.

**Graphical abstract:**

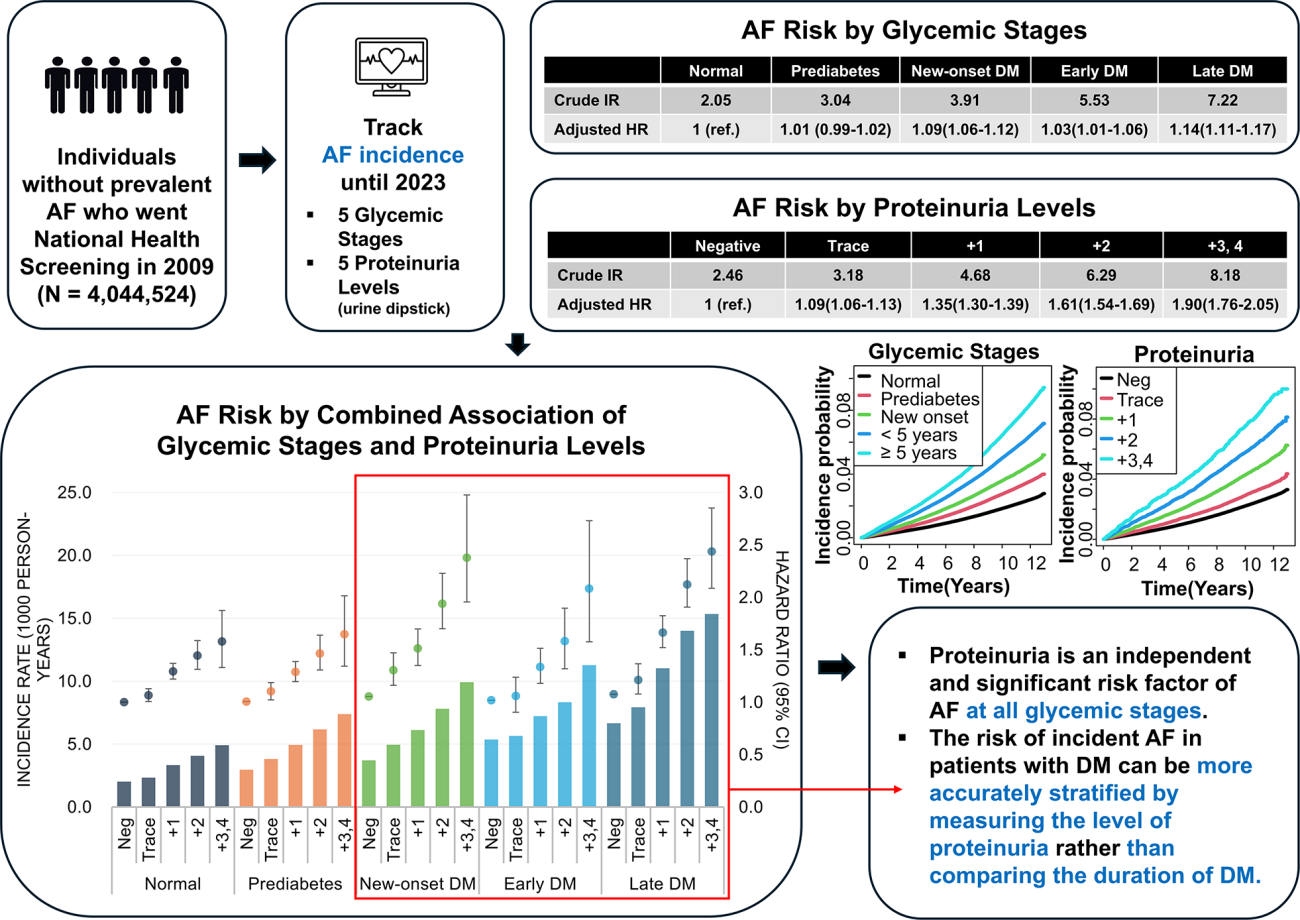

**Supplementary Information:**

The online version contains supplementary material available at 10.1186/s12933-025-02590-2.

## Background

Atrial Fibrillation (AF) is the most prevalent sustained cardiac arrhythmia with numerous recognized risk factors for its development [1, 2]. Proteinuria and diabetes mellitus (DM) are two established cardiovascular risk factors independently associated with an increased risk of AF. Proteinuria is a well-recognized biomarker of chronic kidney disease (CKD) and a predictor of CKD progression [3, 4]. Previous large cohort studies using albumin-to-creatinine ratio (ACR) or urine dipstick tests have shown that proteinuria alone is independently associated with a higher risk of AF, with a greater level of proteinuria correlating with an even higher risk [5–8]. Similarly, several studies have demonstrated an association between DM and the risk of AF, with an approximately 40% increase in the risk of AF in patients [9–11]. Furthermore, the risk is further increased in individuals with a longer duration of DM or poor glycemic control, indicating a cumulative effect of hyperglycemia on AF risk [12, 13].

The association between proteinuria and increased risk of incident AF has been observed in both DM and non-DM populations [14, 15]. Proteinuria and DM are closely related because diabetic nephropathy is a classic microvascular complication of DM, and proteinuria is one of its earliest manifestations [16]. Indeed, proteinuria can be regarded as a manifestation of end-organ damage in patients with DM. Additionally, the development and severity of diabetic nephropathy are highly influenced by the duration and control of hyperglycemia [17]. Prediabetes is also associated with the development of diabetic nephropathy, and proteinuria can be detected before a decline in the glomerular filtration rate (GFR) [18]. However, the relationship between proteinuria and DM, with respect to the risk of incident AF, remains unclear.

Using nationwide population-based health checkup data in Korea, we aimed to investigate the relationship between proteinuria and the risk of incident AF across glycemic stages.

## Methods

### Data source

This study utilized data obtained from the Korean National Health Insurance Database (NHID), a nationwide claims database constructed by the National Health Insurance Service (NHIS). The NHID includes the healthcare utilization database, which consists of a variety of information, including healthcare system usage, prescription records, and diagnoses based on the Korean Standard Classification of Diseases (KCD) and International Classification of Diseases, 10th Revision, Clinical Modification (ICD-10-CM) [19]. It also includes a national health screening database covering health checkup data such as lifestyle questionnaires, anthropometric measurements, and laboratory data [19]. All Koreans aged 20 years or older are recommended to undergo free general health screening every two years.

This study adhered to the Declarations of Helsinki and Istanbul and received an exemption from the Institutional Review Board (IRB) of Seoul National University Hospital (IRB no. 2408-098-1562). Informed consent was not obtained because the database was anonymized. The utilization of the NHID for 2009–2023 was approved in 2024.

### Study population and design

**Fig. 1:**
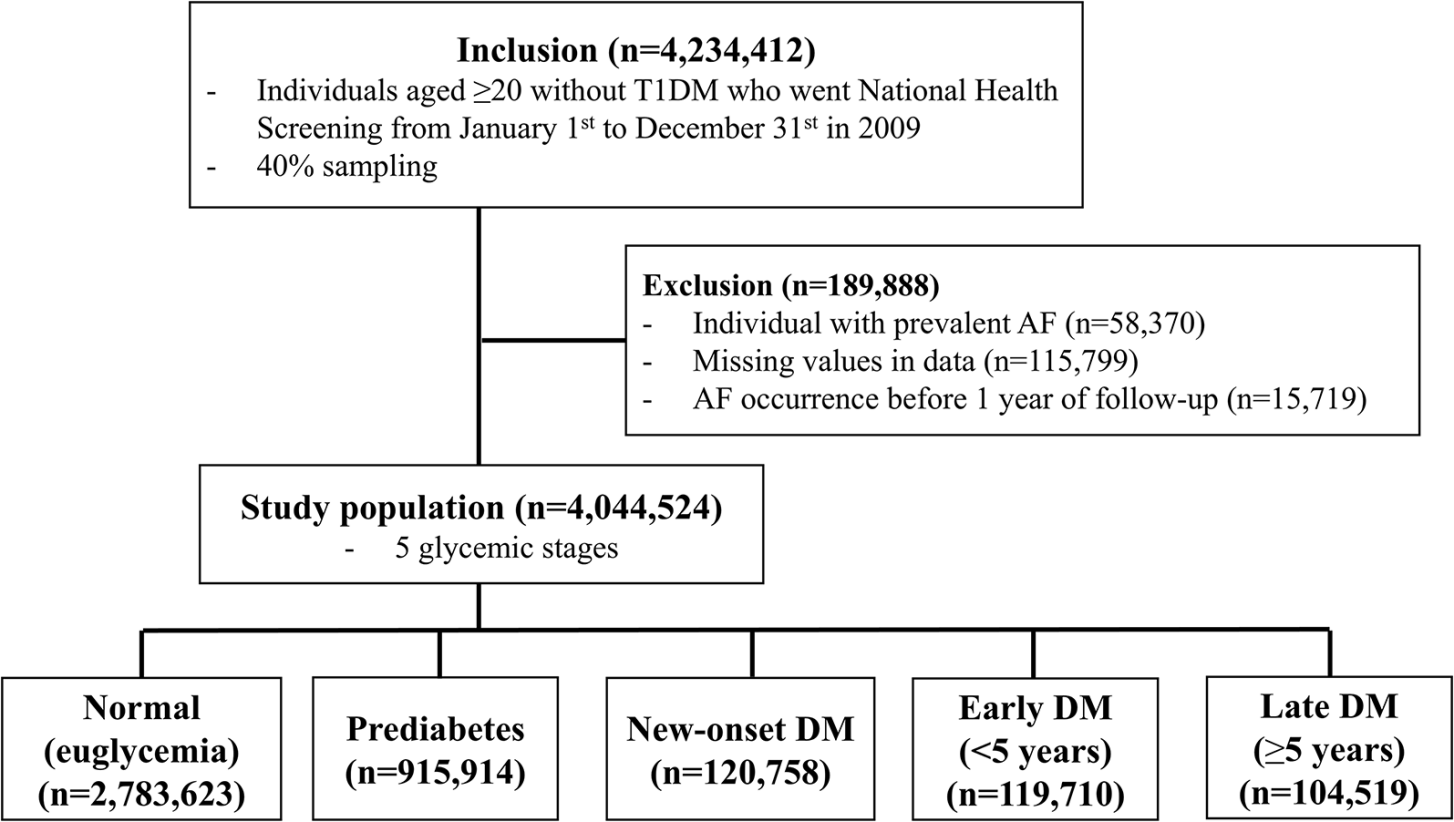
Study population and design. T1DM: type 1 diabetes mellitus; AF: atrial fibrillation; DM: diabetes mellitus

Figure 1 illustrates the study population and design. Individuals aged 20 years or older without type 1 DM who underwent National Health Screening from January 1st to December 31st, 2009, were initially pooled from the database, and 40% of them were randomly sampled (*n* = 4,234,412). Patients with prevalent AF at the time of screening (*n* = 58,370), missing data values (*n* = 115,799), and AF occurrence within 1 year of follow-up (15,719) were excluded, resulting in a study population of 4,044,524 individuals. They were further categorized into five glycemic stages: normal (euglycemia), prediabetes, new-onset DM, early DM (diagnosed < 5 years), and late DM (diagnosed ≥ 5 years). Prediabetes was defined as impaired fasting glucose (fasting glucose level of 100–126 mg/dL), and new-onset DM was defined as DM first diagnosed at the time of screening. Urine dipstick test results were used to estimate the level of proteinuria and categorized as negative, trace, 1+, 2+, or 3+/4+ [8]. AF was defined as at least one diagnosis according to the corresponding ICD-10-CM code (I48) during hospitalization, more than twice at an outpatient clinic, or once in the case of death. The incidence of AF was tracked until December 31st, 2023, or until exclusion from the NHIS as a result of immigration or death, and was compared across glycemic stages and proteinuria levels.

### Covariates

Sociodemographic data and anthropometric measurements were collected, including age, sex, income (in quartiles), body mass index (BMI), waist circumference, smoking status, drinking habits, and physical activity. Obesity was defined as BMI of 25 kg/m^2^ or higher, and abdominal obesity was defined as a waist circumference of 90 cm or higher in men and 85 cm or higher in women [20]. Non-drinker, mild drinker, and heavy drinker were defined as a person with no alcohol intake, less than 30 g of alcohol per day, and 30 g or more alcohol intake per day, respectively. Regular exercise was defined as mid-term exercise for 5 days or more or vigorous exercise for 3 days or more per week. Systolic and diastolic blood pressures were recorded. Comorbidities, including hypertension, dyslipidemia, CKD, DM, heart failure, history of myocardial infarction (MI), and history of ischemic stroke, were recorded. Diabetic neuropathy and diabetic retinopathy were recorded to assess the rate of microvascular complications and to further reflect the glycemic controlled status and contribution of diabetic nephropathy to proteinuria. The detailed definitions of AF, DM, and other comorbidities used in this study are presented in Additional file 1: Table S1. Laboratory test results included urine dipstick test for proteinuria, fasting glucose, total cholesterol, high-density lipoprotein cholesterol (HDL-C), low-density lipoprotein cholesterol (LDL-C), triglycerides, and estimated glomerular filtration rate (eGFR).

### Statistical analysis

Continuous variables following a normal distribution were presented as mean ± standard deviation (SD), and those not following a normal distribution were presented as geometric mean with 95% confidence interval (CI). Categorical variables were presented as numbers and percentages. Group comparisons of baseline characteristics were conducted using one-way analysis of variance (ANOVA), Kruskal–Wallis test, or chi-square test. Changes in baseline characteristics by the glycemic stages were assessed using the p-for-trend. The incidence rate of AF was calculated as the number of new AF events per 1,000 person-years. Kaplan-Meier survival curves of cumulative AF incidence were created and compared using the log-rank test. Cox proportional hazards regression analyses were conducted to determine the hazard ratios (HR) with 95% confidence intervals (CI). Stepwisely, six different Cox Proportional-Hazards models were generated by varying corrected covariates as follows: (i) unadjusted (Model 1); (ii) age and sex (Model 2); (iii) adding income quartile, BMI, smoking status, drinking habit, physical activity, hypertension, dyslipidemia, and CKD on Model 2 (Model 3); (iv) adding heart failure, history of MI, and history of ischemic stroke on Model 3 (Model 4); (v) adding diabetic neuropathy and diabetic retinopathy on Model 3 (Model 5); and (vi) adding diabetic neuropathy and diabetic retinopathy on Model 4 (Model 6). The main analyses were performed using Model 4, and sensitivity analyses were performed using Models 5 and 6, except for the proteinuria level comparison. Harrell’s C-index was calculated to assess the discriminatory power of the models. A competing risk analysis using the Fine-Gray subdistribution hazard model was additionally conducted with all-cause death as a competing risk. Statistical significance was determined for two-tailed p-values < 0.05. Data were analyzed using SAS version 9.4 (SAS Institute, Cary, North Carolina, USA).

## Results

Table 1 presents the baseline characteristics of the study population. The median follow-up time of the population was 12.3 (interquartile range 12.1–12.6) years. A total of 4,044,524 individuals with a mean age of 47 ± 14 years (44.8% females) were included. The five glycemic stages accounted for 69% (2,783,623) of the total population for normal (euglycemia), 23% (*n* = 915,914) for prediabetes, 3.0% (*n* = 120,758) for new-onset DM, 3.0% (*n* = 119,710) for early DM, and 2.6% (*n* = 104,519) for late DM. The mean age gradually increased from the normal group (45 years) to the late DM group (62 years, p-for-trend < 0.001). The normal group had a significantly lower proportion of individuals with obesity and abdominal obesity than the prediabetic (both *p* < 0.001) and DM groups (all *p* < 0.001). The proportions of nonsmokers, nondrinkers, and individuals who exercised regularly were higher in the early and late DM groups than in the normal, prediabetes, and new-onset DM groups. The proportion of individuals with comorbidities, including hypertension, dyslipidemia, CKD, heart failure, history of MI, and history of ischemic stroke, continuously increased from the normal to late DM groups (all p-for-trend < 0.001). The number of individuals with diabetic retinopathy and neuropathy dramatically increased from the normal group to the late DM group (both p-for-trend < 0.001). A small number of participants with diabetic retinopathy and neuropathy were observed in the normal group (0.1% and 0.2%, respectively) and the prediabetes group (0.2% and 0.3%, respectively). Individuals without proteinuria accounted for 95% of the total population, and those with proteinuria detected by urine dipstick test results of trace 1+, 2+, and 3+/4 + accounted for 2.3%, 1.67%, 0.6%, and 0.2% of the population, respectively. The percentage of individuals without proteinuria gradually decreased from the normal group to the late DM group (p-for-trend < 0.001).

**Table 1:** Baseline characteristics of the study population

*n* (%)	Total	Glycemic stages					*p*-for-trends
		Normal glucose metabolism	Prediabetes	New-onset DM	Early DM	Late DM	
	4,044,524	2,783,623 (69%)	915,914 (23%)	120,758 (3.0%)	119,710 (3.0%)	104,519 (2.6%)	
Age	46.9 ± 14.0	44.7 ± 13.8	49.5 ± 13.2	51.7 ± 12.6	58.2 ± 11.1	61.7 ± 9.9	< 0.0001
Female sex	1,811,575 (45%)	1,335,638 (48%)	344,214 (38%)	34,260 (28%)	49,963 (42%)	47,500 (46%)	< 0.0001
Income, Lowest Q1	760,820 (19%)	525,177 (19%)	167,204 (18%)	24,432 (20%)	23,824 (20%)	20,183 (19%)	< 0.0001
Obesity (BMI ≥ 25)	1,319,064 (33%)	784,451 (28%)	367,965 (40%)	58,486 (48%)	62,644 (52%)	45,518 (44%)	< 0.0001
Abdominal obesity	787,175 (20%)	429,055 (15%)	227,847 (25%)	40,538 (34%)	49,890 (42%)	39,845 (38%)	< 0.0001
Smoking							
Never	2,392,716 (59%)	1,706,026 (61%)	496,468 (54%)	55,797 (46%)	68,763 (57%)	65,662 (63%)	< 0.0001
Former	578,373 (14%)	353,962 (13%)	160,882 (18%)	22,292 (19%)	22,485 (19%)	18,752 (18%)	< 0.0001
Current	1,073,435 (27%)	723,635 (26%)	258,564 (28%)	42,669 (35%)	28,462 (24%)	20,105 (19%)	< 0.0001
Drinking							
Non	2,079,533 (51%)	1,451,153 (52%)	433,532 (47%)	52,149 (43%)	72,456 (61%)	70,243 (67%)	< 0.0001
Mild	1,643,042 (41%)	1,141,660 (41%)	386,227 (42%)	51,420 (43%)	36,545 (31%)	27,190 (26%)	< 0.0001
Heavy	321,949 (8.0%)	190,810 (6.9%)	96,155 (11%)	17,189 (14%)	10,709 (9.0%)	7,086 (6.8%)	< 0.0001
Regular exercise	728,006 (18%)	479,351 (17%)	173,593 (19%)	22,872 (19%)	26,654 (22%)	25,536 (24%)	< 0.0001
Hypertension	1,070,321 (26%)	555,667 (20%)	311,264 (34%)	54,069 (45%)	77,176 (64%)	72,145 (69%)	< 0.0001
Dyslipidemia	723,876 (18%)	381,970 (13%)	201,558 (22%)	34,082 (28%)	57,292 (48%)	48,974 (47%)	< 0.0001
Chronic kidney disease	273,511 (6.8%)	160,251 (5.8%)	71,064 (7.8%)	10,314 (8.5%)	13,556 (11%)	18,326 (18%)	< 0.0001
Heart failure	19,105 (0.5%)	9,703 (0.4%)	4,817 (0.5%)	804 (0.7%)	1,980 (1.7%)	1,801 (1.7%)	< 0.0001
Previous myocardial infarction	56,055 (1.4%)	29,879 (1.1%)	14,249 (1.6%)	2,150 (1.8%)	4,267 (3.6%)	5,510 (5.3%)	< 0.0001
Previous ischemic stroke	141,622 (3.5%)	73,229 (2.6%)	35,449 (3.9%)	5,440 (4.5%)	11,825 (9.9%)	15,679 (15%)	< 0.0001
Diabetic retinopathy	39,416 (1.0%)	3,748 (0.1%)	2,020 (0.2%)	1,261 (1.0%)	9,967 (8.3%)	22,420 (21%)	< 0.0001
Diabetic neuropathy	65,018 (1.6%)	5,410 (0.2%)	3,425 (0.4%)	1,834 (1.5%)	20,949 (18%)	33,400 (32%)	< 0.0001
Proteinuria							
Negative	3,852,974 (95%)	2,674,628 (96%)	870,724 (95%)	110,163 (91%)	107,415 (90%)	90,044 (86%)	< 0.0001
Trace	91,593 (2.3%)	56,703 (2.0%)	22,000 (2.4%)	4,156 (3.4%)	4,540 (3.8%)	4,194 (4.0%)	< 0.0001
+ 1	67,532 (1.7%)	37,062 (1.3%)	16,138 (1.8%)	4,159 (3.4%)	4,746 (4.0%)	5,427 (5.2%)	< 0.0001
+ 2	24,877 (0.6%)	12,089 (0.4%)	5,483 (0.6%)	1,765 (1.5%)	2,198 (1.8%)	3,342 (3.2%)	< 0.0001
+ 3,4	7,548 (0.2%)	3,141 (0.1%)	1,569 (0.2%)	515 (0.4%)	811 (0.7%)	1,512 (1.5%)	< 0.0001
Fasting glucose, mg/dL	97.2 ± 23.7	87.5 ± 7.7	107.8 ± 6.6	154.7 ± 42.2	138.6 ± 50.9	147.6 ± 54.4	< 0.0001
eGFR, mL/min/1.73m^2^	87.7 ± 45.3	89.1 ± 49.1	85.2 ± 35.2	85.6 ± 35.9	84.3 ± 37.0	80.5 ± 35.9	< 0.0001
Total cholesterol, mg/dL	195.1 ± 36.9	192.5 ± 35.5	201.9 ± 37.6	207.6 ± 42.1	195.8 ± 42.5	188.8 ± 40.8	< 0.0001
HDL-C, mg/dL	56.1 ± 28.1	56.8 ± 27.9	55.5 ± 28.2	53.4 ± 26.7	52.1 ± 30.2	52.0 ± 31.5	< 0.0001
LDL-C, mg/dL	113.5 ± 38.7	112.5 ± 37.9	117.6 ± 38.9	116.1 ± 43.6	110.1 ± 42.9	106.4 ± 42.2	0.655
* Triglyceride, mg/dL	112.6 (112.5, 112.7)	104.3 (104.3, 104.4)	127.5 (127.4, 127.7)	160.8 (160.2, 161.4)	149.3 (148.8, 149.8)	138.3 (137.8, 138.8)	< 0.0001

*Geometric mean (95% C.I)

DM: diabetes mellitus; BMI: body-mass index; eGFR: estimated glomerular filtration rate; HDL-C: high-density lipoprotein cholesterol; LDL-C: low-density lipoprotein cholesterol

### Incident AF risks compared by different glycemic stages and proteinuria level

**Fig. 2:**
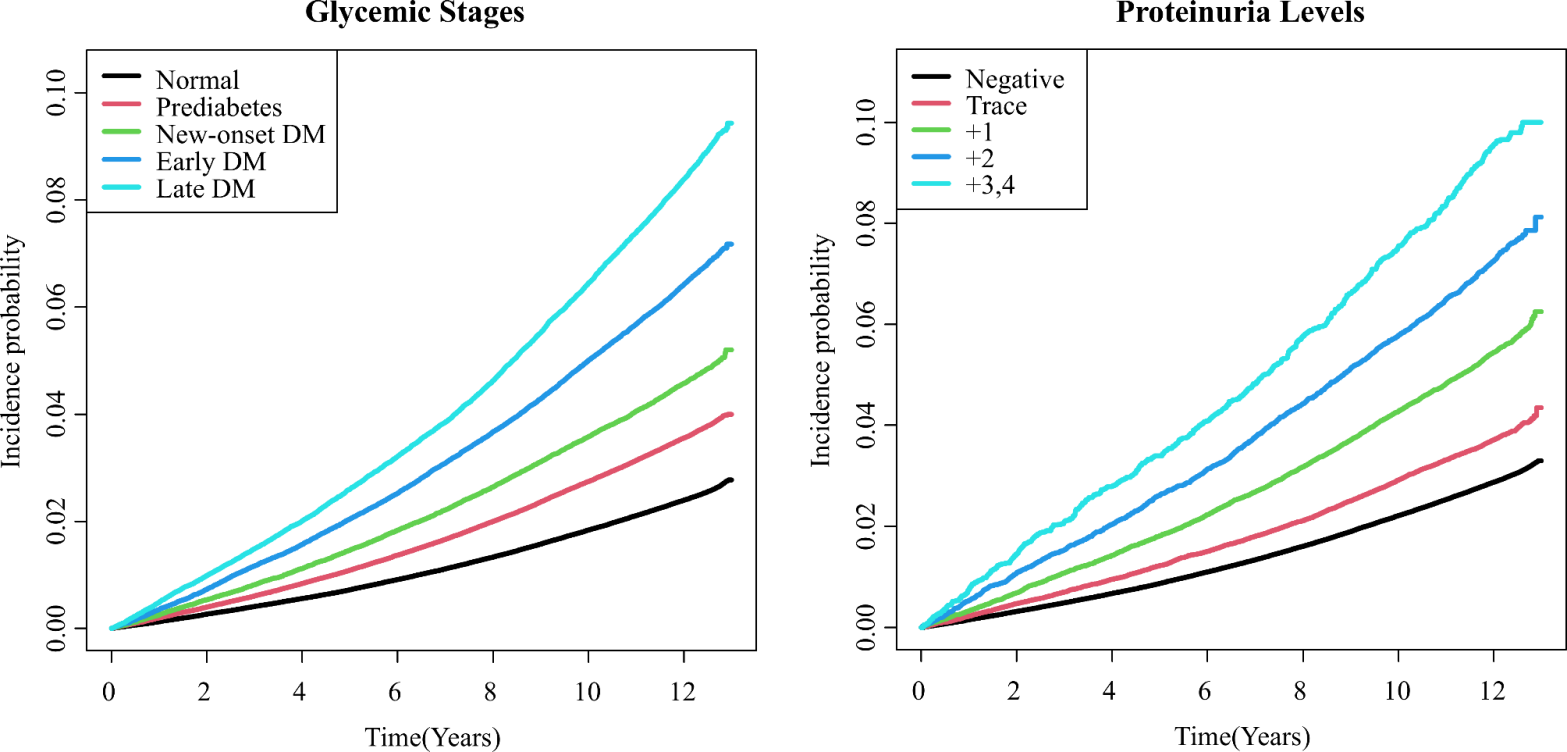
Kaplan-Meier curves for AF incidence corresponding to glycemic stages and proteinuria level. DM: diabetes mellitus; Prediabetes: impaired fasting glucose (fasting glucose level from 100 to 126 mg/dL); New-onset DM: DM first diagnosed at the time of screening; Early DM: DM duration after diagnosis < 5 years; Late DM: DM duration after diagnosis ≥ 5 years

The incidence rates of AF across different glycemic stages and proteinuria levels are shown in Table 2. The crude incidence rates of AF per 1000 person-years showed a trend of heightened risk across the following glycemic stages: 2.05 (normal), 3.04 (prediabetes), 3.91 (new-onset DM), 5.53 (early DM), and 7.22 (late DM). Similarly, AF incidence rates exhibited a graded association with the degree of proteinuria level: 2.46 (negative), 3.18 (trace), 4.68 (1+), 6.29 (2+), and 8.18 (3+/4+). The Kaplan-Meier curves corresponding to the glycemic stages and proteinuria levels are presented in Fig. 2.

**Table 2:** Incidence rates of AF across glycemic stages and proteinuria levels

Groups	AF Incidence Rate, 1000 PY	Hazard Ratio (95% CI)					
		Model 1	Model 2	Model 3	Model 4	Model 5	Model 6
Glycemic Stages							
Normal	2.05	1 (ref.)	1 (ref.)	1 (ref.)	1 (ref.)	1 (ref.)	1 (ref.)
Prediabetes	3.04	1.49 (1.47, 1.51)	1.08 (1.07, 1.10)	1.00 (0.99, 1.02)	1.01 (0.99, 1.02)	1.00 (0.99, 1.02)	1.01 (0.99, 1.02)
New-onset DM	3.91	1.93 (1.87, 1.98)	1.22 (1.19, 1.26)	1.08 (1.05, 1.11)	1.09 (1.06, 1.12)	1.08 (1.05, 1.11)	1.08 (1.05, 1.11)
Early DM	5.53	2.73 (2.66, 2.79)	1.23 (1.20, 1.26)	1.04 (1.02, 1.07)	1.03 (1.01, 1.06)	1.01 (0.99, 1.04)	1.01 (0.98, 1.03)
Late DM	7.22	3.60 (3.52, 3.69)	1.35 (1.32, 1.38)	1.16 (1.14, 1.19)	1.14 (1.11, 1.17)	1.09 (1.06, 1.12)	1.08 (1.05, 1.11)
p-value		< 0.0001	< 0.0001	< 0.0001	< 0.0001	< 0.0001	< 0.0001
C-index		0.578	0.794	0.800	0.800	0.801	0.801
Proteinuria levels							
Negative	2.46	1 (ref.)	1 (ref.)	1 (ref.)	1 (ref.)		
Trace	3.18	1.30 (1.25, 1.34)	1.15 (1.12, 1.19)	1.10 (1.06, 1.13)	1.09 (1.06, 1.13)		
+ 1	4.68	1.92 (1.86, 1.98)	1.48 (1.44, 1.53)	1.35 (1.31, 1.40)	1.35 (1.30, 1.39)		
+ 2	6.29	2.59 (2.47, 2.72)	1.86 (1.77, 1.95)	1.62 (1.55, 1.70)	1.61 (1.54, 1.69)		
+ 3,4	8.18	3.40 (3.15, 3.68)	2.26 (2.09, 2.44)	1.92 (1.78, 2.07)	1.90 (1.76, 2.05)		
p-value		< 0.0001	< 0.0001	< 0.0001	< 0.0001		
C-index		0.516	0.794	0.800	0.800		

Model 1: non-adjusted; Model 2: age, sex; Model 3: age, sex, income quartile, body-mass index (BMI), smoking status, drinking habit, physical activity, hypertension, dyslipidemia, and chronic kidney disease; Model 4: age, sex, income quartile, BMI, smoking status, drinking habit, physical activity, hypertension, dyslipidemia, chronic kidney disease, heart failure, history of myocardial infarction, and history of ischemic stroke; Model 5: Model 3 + diabetic retinopathy and diabetic neuropathy; Model 6: Model 4 + diabetic retinopathy and diabetic neuropathy

AF: atrial fibrillation; PY: person-years; 95% CI: 95% confidence interval; ref.: reference value for hazard ratio; DM: diabetes mellitus; C-index: Harrell’s C-index

When adjusted for age, sex, income quartile, BMI, smoking status, drinking habits, physical activity, hypertension, dyslipidemia, chronic kidney disease, heart failure, history of myocardial infarction, and history of ischemic stroke (Model 4), the differences in the incidence rates were attenuated with weakened statistical significance. In Model 4, the incidence rate of AF in the prediabetes group was similar to that of the normal group (HR 1.01, 95% CI 0.99–1.02), while incidence rates were significantly higher in the new-onset DM, early DM, and late DM groups (HR 1.09, 1.03, and 1.14, 95% CI 1.06–1.12, 1.01–1.06, and 1.11–1.17, respectively) compared to the normal group. HRs and their statistical significance were further attenuated to 1.08, 1.01, 1.08 in the new-onset DM, early DM, and late DM groups, respectively, after additional adjustments for diabetic neuropathy and retinopathy (Model 6). In contrast, HRs by proteinuria level in Model 4 exhibited more distinct differences, with HR of 1.09, 1.35, 1.61, and 1.90 in the trace, 1+, 2+, and 3+/4 + groups, respectively. Harrell’s C-index for Model 4 in each case was approximately 0.8, indicating good discrimination.

### Interactions of glycemic stages and proteinuria level with the risk of incident AF

We further compared the HRs of the AF incidence rate by proteinuria level within each glycemic stage (Table 3). Higher crude incidence rates of AF with increasing proteinuria were observed at all glycemic stages. The corresponding Kaplan-Meier curves are presented in Additional file 1: Figure S1.

**Table 3:** Hazard ratios of AF incidence rate by proteinuria level within each glycemic stage

Glycemic stages	Proteinuria	AF Incidence Rate, 1000 PY	Hazard Ratio (95% CI)					
			Model 1	Model 2	Model 3	Model 4	Model 5	Model 6
Normal	Neg	2.01	1 (ref.)	1 (ref.)	1 (ref.)	1 (ref.)	1 (ref.)	1 (ref.)
	Trace	2.33	1.16 (1.10, 1.22)	1.11 (1.05, 1.16)	1.07 (1.02, 1.12)	1.06 (1.01, 1.12)	1.07 (1.02, 1.12)	1.06 (1.01, 1.12)
	+ 1	3.33	1.66 (1.58, 1.75)	1.40 (1.33, 1.47)	1.29 (1.23, 1.36)	1.29 (1.23, 1.36)	1.29 (1.23, 1.36)	1.29 (1.23, 1.36)
	+ 2	4.06	2.03 (1.87, 2.20)	1.62 (1.50, 1.76)	1.45 (1.33, 1.57)	1.44 (1.33, 1.57)	1.45 (1.33, 1.57)	1.44 (1.33, 1.57)
	+ 3,4	4.91	2.46 (2.12, 2.86)	1.81 (1.56, 2.10)	1.58 (1.36, 1.84)	1.58 (1.36, 1.83)	1.58 (1.36, 1.84)	1.58 (1.36, 1.83)
Prediabetes	Neg	2.96	1.48 (1.46, 1.50)	1.08 (1.06, 1.09)	1.00 (0.99, 1.02)	1.00 (0.99, 1.02)	1.00 (0.99, 1.02)	1.00 (0.99, 1.02)
	Trace	3.82	1.91 (1.79, 2.03)	1.24 (1.16, 1.32)	1.10 (1.03, 1.18)	1.10 (1.03, 1.17)	1.10 (1.03, 1.17)	1.10 (1.03, 1.17)
	+ 1	4.94	2.48 (2.32, 2.64)	1.49 (1.39, 1.59)	1.29 (1.20, 1.37)	1.29 (1.21, 1.38)	1.29 (1.20, 1.37)	1.29 (1.21, 1.37)
	+ 2	6.17	3.10 (2.80, 3.43)	1.75 (1.58, 1.94)	1.47 (1.33, 1.62)	1.46 (1.32, 1.62)	1.47 (1.32, 1.62)	1.46 (1.32, 1.62)
	+ 3,4	7.38	3.72 (3.13, 4.43)	2.02 (1.70, 2.41)	1.64 (1.38, 1.96)	1.65 (1.38, 1.96)	1.64 (1.38, 1.95)	1.64 (1.38, 1.96)
New-onset DM	Neg	3.71	1.86 (1.80, 1.91)	1.18 (1.15, 1.22)	1.05 (1.02, 1.08)	1.05 (1.02, 1.09)	1.05 (1.02, 1.08)	1.05 (1.02, 1.08)
	Trace	4.95	2.49 (2.19, 2.83)	1.52 (1.33, 1.72)	1.30 (1.14, 1.48)	1.30 (1.15, 1.48)	1.29 (1.14, 1.47)	1.30 (1.14, 1.48)
	+ 1	6.11	3.08 (2.74, 3.46)	1.81 (1.61, 2.03)	1.51 (1.34, 1.70)	1.51 (1.34, 1.70)	1.50 (1.34, 1.69)	1.50 (1.34, 1.69)
	+ 2	7.80	3.96 (3.37, 4.65)	2.36 (2.01, 2.78)	1.93 (1.64, 2.27)	1.94 (1.65, 2.28)	1.92 (1.63, 2.26)	1.93 (1.64, 2.26)
	+ 3,4	9.93	5.09 (3.89, 6.66)	2.96 (2.26, 3.87)	2.39 (1.83, 3.13)	2.38 (1.82, 3.11)	2.36 (1.80, 3.09)	2.35 (1.80, 3.08)
Early DM	Neg	5.36	2.69 (2.62, 2.76)	1.21 (1.18, 1.24)	1.03 (1.00, 1.06)	1.02 (0.99, 1.04)	1.00 (0.97, 1.03)	1.00 (0.97, 1.02)
	Trace	5.65	2.84 (2.53, 3.19)	1.30 (1.15, 1.46)	1.07 (0.95, 1.20)	1.06 (0.94, 1.19)	1.04 (0.92, 1.17)	1.03 (0.92, 1.16)
	+ 1	7.22	3.65 (3.30, 4.04)	1.68 (1.51, 1.86)	1.35 (1.22, 1.49)	1.34 (1.20, 1.48)	1.31 (1.18, 1.45)	1.30 (1.18, 1.44)
	+ 2	8.32	4.23 (3.67, 4.88)	2.05 (1.78, 2.36)	1.60 (1.39, 1.84)	1.58 (1.37, 1.82)	1.54 (1.34, 1.78)	1.54 (1.33, 1.77)
	+ 3,4	11.27	5.78 (4.70, 7.10)	2.77 (2.26, 3.41)	2.12 (1.73, 2.61)	2.08 (1.70, 2.56)	2.05 (1.67, 2.52)	2.02 (1.64, 2.48)
Late DM	Neg	6.65	3.37 (3.28, 3.45)	1.26 (1.22, 1.29)	1.10 (1.07, 1.12)	1.08 (1.05, 1.10)	1.04 (1.01, 1.07)	1.03 (1.00, 1.06)
	Trace	7.93	4.04 (3.64, 4.49)	1.49 (1.34, 1.66)	1.25 (1.12, 1.38)	1.21 (1.09, 1.35)	1.18 (1.06, 1.31)	1.16 (1.04, 1.28)
	+ 1	11.03	5.68 (5.24, 6.15)	2.08 (1.92, 2.25)	1.70 (1.57, 1.84)	1.66 (1.54, 1.80)	1.60 (1.48, 1.74)	1.58 (1.46, 1.72)
	+ 2	14.00	7.30 (6.65, 8.02)	2.74 (2.49, 3.00)	2.20 (2.00, 2.41)	2.12 (1.93, 2.33)	2.06 (1.87, 2.26)	2.01 (1.82, 2.21)
	+ 3,4	15.33	8.13 (7.09, 9.32)	3.23 (2.81, 3.70)	2.52 (2.20, 2.89)	2.44 (2.12, 2.80)	2.33 (2.03, 2.68)	2.28 (1.99, 2.62)
p-value			< 0.0001	< 0.0001	< 0.0001	< 0.0001	< 0.0001	< 0.0001
C-index			0.583	0.795	0.800	0.802	0.800	0.802

Model 1: non-adjusted; Model 2: age, sex; Model 3: age, sex, income quartile, body-mass index (BMI), smoking status, drinking habit, physical activity, hypertension, dyslipidemia, and chronic kidney disease; Model 4: age, sex, income quartile, BMI, smoking status, drinking habit, physical activity, hypertension, dyslipidemia, chronic kidney disease, heart failure, history of myocardial infarction, and history of ischemic stroke; Model 5: Model 3 + diabetic retinopathy and diabetic neuropathy; Model 6: Model 4 + diabetic retinopathy and diabetic neuropathy

AF: atrial fibrillation; PY: person-years; 95% CI: 95% confidence interval; ref.: reference value for hazard ratio; Neg: negative urine dipstick test; DM: diabetes mellitus; C-index: Harrell’s C-index

Adjusted for covariates in the Cox regression Model 4, a heightened risk of incident AF associated with higher proteinuria levels was observed in all glycemic stages. The adjusted HRs for negative urine dipstick test results were 1 (reference), 1.00, 1.05, 1.02, and 1.08, and for urine dipstick test results of 3+/4 were 1.58, 1.65, 2.38, 2.08, and 2.44 in the normal, prediabetes, new-onset DM, early DM, and late DM groups, respectively.

**Fig. 3:**
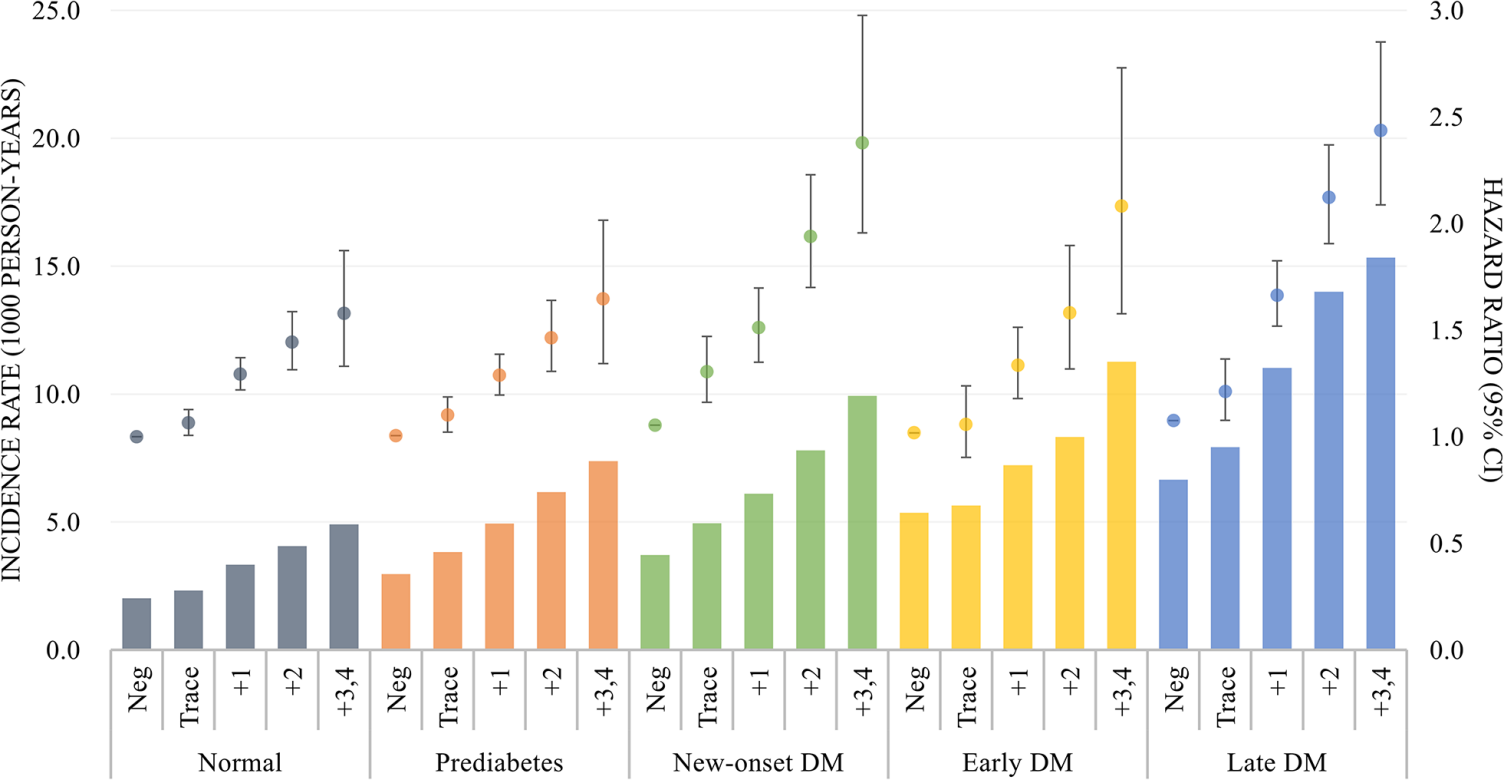
AF incidence rates and hazard ratios in Cox Proportional-Hazards Regression Model 4. DM: diabetes mellitus; Prediabetes: impaired fasting glucose (fasting glucose level from 100 to 126 mg/dL); New-onset DM: DM first diagnosed at the time of screening; Early DM: DM duration after diagnosis < 5 years; Late DM: DM duration after diagnosis ≥ 5 years

Figure 3 shows the incidence rates of AF and their HRs in Model 4. The Cox regression model with interaction terms revealed that the HRs for the urine dipstick test 3+/4 + and the interaction effect for increasing proteinuria were higher in new-onset DM, early DM, and late DM individuals than in normal and prediabetic individuals (p-for-interaction < 0.001). However, among the new-onset, early, and late DM groups, there was no statistical difference in the elevated risk of AF according to proteinuria level (adjusted HRs for urine dipstick test results of 3+/4 with reference to negative urine dipstick test were 2.26, 2.04, and 2.28 in the new-onset, early, and late DM groups, respectively; Additional file 1: Table S2). When the models were further adjusted for diabetic neuropathy and retinopathy, the HRs and interaction effects remained significant, although the HRs were more attenuated with longer durations of DM.

### Competing risk analysis

Competing risk models were further constructed using the same covariates, with all-cause death considered as a competing risk (Additional file 2: Tables S3 and S4). In Model 4, only the new-onset DM and late DM groups showed a statistically significant higher risk of AF compared to the normal group (HR: 1.04, 95% CI: 1.02–1.08, and HR: 1.07, 95% CI: 1.04–1.09, respectively). The prediabetes and early DM groups had HRs of 1.01 (95% CI: 1.00–1.02) and 1.00 (95% CI: 0.97–1.02), respectively. Consistent with the prior analysis, HRs by proteinuria level in Model 4 showed progressively higher risks, with HRs of 1.06, 1.28, 1.45, and 1.62 for the trace, 1+, 2+, and 3+/4 + groups, respectively.

For AF incidence rates by proteinuria level within each glycemic stage, HRs for negative urine dipstick test results were 1 (reference), 1.01, 1.02, 1.00, and 1.02, while HRs for urine dipstick test results of 3+/4 were 1.45, 1.47, 2.00, 1.75, and 1.79 in the normal, prediabetes, new-onset DM, early DM, and late DM groups, respectively. Cumulative incidence curves and a figure for incidence rates and HRs by proteinuria level within each glycemic stage are presented in Additional file 2: Figures S2, S3, and S4. Interaction analysis consistently revealed that the HRs for increasing proteinuria were consistently higher in the three DM groups compared to the normal and prediabetic groups (p-for-interaction < 0.001).

## Discussion

This study comprehensively compared the risk of incident AF across different glycemic stages and proteinuria levels. The key findings of this study are as follows: (1) DM was significantly associated with the risk of AF; (2) higher proteinuria levels were significantly associated with a higher risk of AF; and (3) the higher risk of AF by graded proteinuria level was similar among individuals with new-onset and established DM, suggesting the importance of measuring the level of proteinuria to accurately measure the cumulative effect of hyperglycemia and stratify AF risk in patients with DM.

These findings provide valuable information for the risk management and early detection of AF in patients at various glycemic stages and proteinuria levels. Indeed, there were distinct differences in baseline characteristics among the different glycemic stage groups. Patients with DM exhibited higher rates of obesity and abdominal obesity than those with prediabetes, which, in turn, showed higher rates than normal individuals. The same pattern was observed for comorbidities, such as hypertension, dyslipidemia, CKD, heart failure, history of MI, and history of stroke. However, unhealthy habits, such as smoking, drinking, and less physical activity, were less prevalent in people with established DM and more common in those with new-onset DM. These findings suggest positive behavioral changes following a DM diagnosis, as supported by several studies on health-related behavioral changes after the diagnosis of non-communicable diseases [21–24]. More individuals with DM presented with proteinuria, and the degree of proteinuria increased with longer DM duration. This is likely due to a higher prevalence of diabetic nephropathy with longer DM duration and an increasing proportion of CKD, diabetic neuropathy, and retinopathy with longer DM duration, corroborating this hypothesis.

The crude incidence rates of AF increased with glycemic stage progression; however, the HRs for AF risk were attenuated and became insignificant when the models were adjusted for several comorbidities. The complex pathophysiology linking DM and AF involves structural and electrical remodeling of the heart induced by oxidative stress and chronic inflammation resulting from hyperglycemia [25]. The cumulative risk of AF in patients with DM is influenced by glycemic control, suggesting that DM does not independently affect AF risk without considering other coexisting metabolic disorders that often share interconnected pathophysiological mechanisms. Furthermore, comparison of AF risks by combined glycemic stages and proteinuria levels showed that the incidence rates in individuals without proteinuria were similar, regardless of the glycemic stage. In contrast, significant differences in the AF risk were observed in patients with substantial proteinuria. This indicates that the increased risk of AF is more pronounced in patients with DM who have developed diabetic nephropathy and related proteinuria, highlighting the compounded effects of hyperglycemia and renal impairment on AF risk.

The association between higher levels of proteinuria and increased AF risk was more pronounced and robust than its association with glycemic stage. In the Cox regression models, urine dipstick test results of trace, 1+, 2+, and 3+/4 + proteinuria were linked to approximately 9%, 35%, 61%, and 90% increase in AF risk, respectively, across the total population. This HR gradient in hazard ratios suggests a cumulative effect of DM on AF risk. As the duration of DM reflects the time elapsed since diagnosis rather than the actual onset of the disease, higher proteinuria levels may more accurately represent the cumulative impact of hyperglycemia. Sensitivity analyses with additional adjustments for two other microvascular complications also attenuated the HRs for AF risk across glycemic stage progression, supporting this interpretation. Furthermore, the association between proteinuria and incident AF was notably stronger in individuals with new-onset and established DM than in those with normal glucose or prediabetic levels.

This interaction effect may indicate that proteinuria unrelated to DM could result from a localized glomerular pathology or systemic diseases that do not share similar risk factors with DM, leading to a weaker association with AF risk. The HRs for high proteinuria levels were similar among the new-onset DM, early DM, and late DM groups, indicating that accurately stratifying the risk of incident AF in patients with DM requires consideration of the controlled status of DM through proteinuria measurement rather than merely comparing DM duration. Competing risk analysis revealed more conservative sub-distribution HRs but consistent results regarding the associations between proteinuria, glycemic stages, and incident AF, supporting the interpretation.

Some cohort studies have revealed that alleviating proteinuria is associated with a lower AF risk than persistent or aggravated proteinuria [7, 8, 15], suggesting that mitigating proteinuria in patients with DM might reduce the risk of AF. In light of our findings, the risk of incident AF in patients with DM could be easily estimated using urine dipstick test results, and clinical strategies for detecting and managing AF in these patients can be tailored based on these results.

### Study limitations

This study has several limitations. First, this study was sensitive to selection bias owing to the use of a nationwide, population-based, retrospective observational database. Although the National Health Screening Program provides free biennial screening to all Koreans, the participation rate in 2009 was 66.0% [26]. Therefore, individuals inclined toward health-related behaviors were more likely to be included in the study population, particularly among patients with DM who regularly attend clinics and have well-controlled DM. Second, the urine dipstick test is semiquantitative, and its diagnostic sensitivity is reported to be approximately 76% [27]. Furthermore, the analysis was based on dipstick results from only one spot of the urine sample, which can introduce high stochastic noise and potentially reduce the significance of the associations. Third, although we analyzed the risk of AF across different DM durations, the controlled status of DM was not considered because of the analyses due to a lack of biomarkers in the database. Fourth, the incidence of AF was derived solely from the claims data, leaving room for undetected, unreported, and misclassified cases. Fifth, a small number of diabetic retinopathy and neuropathy was also observed in euglycemic participants, indicating a degree of misclassification and a potential introduction of bias into the sensitivity analyses. Although the portion of misclassification was small, we limited the use of HR results to support the main findings. Sixth, the models did not adjust for potential confounding effects of medications, such as angiotensin-converting enzyme inhibitors and angiotensin II receptor blockers, which could influence proteinuria levels. Seventh, in competing risk analyses, we could not separate the cases where AF diagnosed with itself being a cause of death due to the lack of specific cause of death data. Finally, there is a generalization issue, given that the dataset consisted only of the Korean population.

## Conclusions

Proteinuria is an independent and significant risk factor for incident AF at all glycemic stages. The risk of incident AF in patients with DM can be stratified by measuring the level of proteinuria rather than comparing the duration of DM. Tailoring clinical strategies to proteinuria level could potentially mitigate this risk, improving patient outcomes. 

## Electronic supplementary material

Below is the link to the electronic supplementary material.

Additional file 1. Table S1. Definitions of variables in the study. Table S2. Hazard ratios of AF incidence rate by proteinuria level within each glycemic stage (negative proteinuria in each glycemic stage as reference). Figure S1. Kaplan-Meier curves for AF incidence corresponding proteinuria level for each glycemic stage.

Additional file 2. Table S3. Incidence rates of AF across glycemic stages and proteinuria levels using competing risk models. Table S4. Hazard ratios of AF incidence rate by proteinuria level within each glycemic stage using competing risk models. Figure S2. Cumulative incidence curves for AF incidence corresponding to glycemic stages and proteinuria level using competing risk models.Figure S3. Cumulative incidence curves for AF incidence corresponding proteinuria level for each glycemic stage using competing risk models. Figure S4. AF incidence rates and hazard ratios in Cox Proportional-Hazards Regression Model 4 with competing risks.

## Data Availability

The data that support the findings of this study are available from NHIS, but restrictions apply to the availability of these data, which were used under license for the current study and therefore are not publicly available. Data are however available from the authors upon reasonable request and with permission of NHIS.

## Abbreviations

ACR: Albumin-to-creatinine ratio
AF: Atrial fibrillation
ANOVA: Analysis of variance
BMI: Body mass index
CI: Confidence interval
CKD: Chronic kidney disease
DM: Diabetes mellitus
eGFR: Estimated glomerular filtration rate
GFR: Glomerular filtration rate
HDL-C: High-density lipoprotein cholesterol
HR: Hazard ratio
ICD-10-CM: International classification of diseases, 10th revision, clinical modification
IRB: Institutional review board
KCD: Korean standard classification of diseases
LDL-C: Low-density lipoprotein cholesterol
MI: Myocardial infarction
NHID: National health insurance database
NHIS: National health insurance service
SD: Standard deviation
