# Supplementary material for: Proteinuria and the risk of Incident atrial fibrillation according to glycemic stages: a nationwide population-based cohort study
Source: Cardiovasc Diabetol. 2025 Jan 24;24:41. doi: 10.1186/s12933-025-02590-2 (PMC11762047; doi:10.1186/s12933-025-02590-2)

Table S1. Definitions of variables in the study.

Variables	ICD-10-CM codes	Definitions
**Comorbidities**		
Type 2 diabetes mellitus	E11-14	One diagnosis and use of anti-diabetic medications for the past year or serum fasting glucose ≥126 mg/dL
Heart failure	I50	One diagnosis for the past year
Hypertension	I10; I11; I12; I13; I15	One diagnosis and use of anti-hypertensive medications for the past year or systolic blood pressure ≥140 mmHg or diastolic blood pressure ≥90 mmHg
Dyslipidemia	E78	One diagnosis and use of anti-hyperlipidemic medications for the past year or serum total cholesterol ≥240 mg/dL
Ischemic stroke	I63; I64	One diagnosis before health checkup
Myocardial infarction	I21; I22	One diagnosis before health checkup
Chronic kidney disease		Estimated glomerular filtration rate <60 mL/min/1.73m^2
Diabetic retinopathy	E104, E114, E124, E134, E144, G590, G632, G990	One diagnosis for the past year
Diabetic neuropathy	H360	One diagnosis for the past year
**Clinical outcome**		
Atrial fibrillation	I48	One diagnosis during hospitalization or more than twice at outpatient clinics for the past year
**Sociodemographic**		
Obesity		BMI ≥25 kg/m^2
Abdominal obesity		Waist circumference ≥90 cm in men or ≥85 cm in women
Drinking		Non: no alcohol intake; Mild: 0 < alcohol intake < 30g/day; Heavy: alcohol intake ≥30g/day
Regular exercise		Mid-term exercise ≥5 days or vigorous exercise ≥3 days in a week

ICD-10-CM: International Classification of Diseases, 10th Revision, Clinical Modification

Table S2. Hazard ratios of AF incidence rate by proteinuria level within each glycemic stage (negative proteinuria in each glycemic stage as reference).

Glycemic stages	Proteinuria	AF Incidence Rate, 1000 PY	Hazard Ratio (95% CI)					
			Model 1	Model 2	Model 3	Model 4	Model 5	Model 6
Normal	Neg	2.01	1 (ref.)	1 (ref.)	1 (ref.)	1 (ref.)	1 (ref.)	1 (ref.)
	Trace	2.33	1.16 (1.10, 1.22)	1.11 (1.05, 1.16)	1.07 (1.02, 1.12)	1.06 (1.01, 1.12)	1.07 (1.02, 1.12)	1.06 (1.01, 1.12)
	+1	3.33	1.66 (1.58, 1.75)	1.40 (1.33, 1.47)	1.29 (1.23, 1.36)	1.29 (1.23, 1.36)	1.29 (1.23, 1.36)	1.29 (1.23, 1.36)
	+2	4.06	2.03 (1.87, 2.20)	1.62 (1.50, 1.76)	1.45 (1.33, 1.57)	1.44 (1.33, 1.57)	1.45 (1.33, 1.57)	1.44 (1.33, 1.57)
	+3,4	4.91	2.46 (2.12, 2.86)	1.81 (1.56, 2.10)	1.58 (1.36, 1.84)	1.58 (1.36, 1.83)	1.58 (1.36, 1.84)	1.58 (1.36, 1.83)
Prediabetes	Neg	2.96	1 (ref.)	1 (ref.)	1 (ref.)	1 (ref.)	1 (ref.)	1 (ref.)
	Trace	3.82	1.29 (1.21, 1.38)	1.15 (1.08, 1.23)	1.10 (1.03, 1.17)	1.10 (1.03, 1.17)	1.10 (1.03, 1.17)	1.10 (1.03, 1.17)
	+1	4.94	1.68 (1.57, 1.79)	1.38 (1.30, 1.48)	1.28 (1.20, 1.37)	1.28 (1.20, 1.37)	1.28 (1.20, 1.37)	1.28 (1.20, 1.37)
	+2	6.17	2.10 (1.90, 2.33)	1.63 (1.47, 1.80)	1.46 (1.32, 1.62)	1.46 (1.32, 1.61)	1.46 (1.32, 1.62)	1.45 (1.31, 1.61)
	+3,4	7.38	2.52 (2.12, 3.00)	1.88 (1.58, 2.24)	1.64 (1.38, 1.95)	1.64 (1.38, 1.95)	1.64 (1.37, 1.95)	1.64 (1.38, 1.95)
New-onset DM	Neg	3.71	1 (ref.)	1 (ref.)	1 (ref.)	1 (ref.)	1 (ref.)	1 (ref.)
	Trace	4.95	1.34 (1.18, 1.53)	1.28 (1.13, 1.46)	1.24 (1.08, 1.41)	1.24 (1.08, 1.41)	1.24 (1.08, 1.41)	1.24 (1.08, 1.41)
	+1	6.11	1.66 (1.47, 1.87)	1.53 (1.36, 1.72)	1.44 (1.27, 1.62)	1.43 (1.27, 1.62)	1.43 (1.27, 1.62)	1.43 (1.27, 1.61)
	+2	7.80	2.13 (1.81, 2.51)	2.00 (1.70, 2.36)	1.84 (1.56, 2.17)	1.84 (1.56, 2.17)	1.83 (1.55, 2.16)	1.83 (1.55, 2.16)
	+3,4	9.93	2.74 (2.09, 3.59)	2.50 (1.91, 3.28)	2.28 (1.74, 2.98)	2.26 (1.72, 2.96)	2.25 (1.72, 2.95)	2.24 (1.71, 2.94)
Early DM	Neg	5.36	1 (ref.)	1 (ref.)	1 (ref.)	1 (ref.)	1 (ref.)	1 (ref.)
	Trace	5.65	1.06 (0.94, 1.19)	1.08 (0.96, 1.21)	1.04 (0.92, 1.17)	1.04 (0.92, 1.17)	1.04 (0.92, 1.17)	1.04 (0.92, 1.17)
	+1	7.22	1.36 (1.22, 1.51)	1.39 (1.25, 1.54)	1.31 (1.18, 1.45)	1.31 (1.18, 1.46)	1.31 (1.18, 1.45)	1.31 (1.18, 1.45)
	+2	8.32	1.57 (1.36, 1.82)	1.70 (1.47, 1.96)	1.55 (1.35, 1.80)	1.55 (1.34, 1.79)	1.54 (1.34, 1.78)	1.54 (1.34, 1.78)
	+3,4	11.27	2.15 (1.75, 2.64)	2.30 (1.87, 2.83)	2.07 (1.68, 2.54)	2.04 (1.66, 2.51)	2.05 (1.66, 2.52)	2.03 (1.65, 2.50)
Late DM	Neg	6.65	1 (ref.)	1 (ref.)	1 (ref.)	1 (ref.)	1 (ref.)	1 (ref.)
	Trace	7.93	1.20 (1.08, 1.34)	1.19 (1.07, 1.32)	1.14 (1.02, 1.27)	1.13 (1.01, 1.25)	1.13 (1.02, 1.26)	1.12 (1.01, 1.25)
	+1	11.03	1.68 (1.55, 1.83)	1.66 (1.52, 1.80)	1.55 (1.43, 1.69)	1.55 (1.42, 1.68)	1.54 (1.42, 1.68)	1.54 (1.42, 1.67)
	+2	14.00	2.17 (1.97, 2.39)	2.18 (1.98, 2.40)	2.00 (1.82, 2.21)	1.97 (1.79, 2.17)	1.98 (1.80, 2.18)	1.95 (1.77, 2.15)
	+3,4	15.33	2.41 (2.10, 2.77)	2.57 (2.24, 2.95)	2.30 (2.00, 2.64)	2.28 (1.99, 2.62)	2.25 (1.96, 2.59)	2.22 (1.93, 2.56)
p-value			<.0001	<.0001	<.0001	<.0001	<.0001	<.0001
C-index			0.583	0.795	0.800	0.802	0.800	0.802

Model 1: non-adjusted; Model 2: age, sex; Model 3: age, sex, income quartile, body-mass index (BMI), smoking status, drinking habit, physical activity, hypertension, dyslipidemia, and chronic kidney disease; Model 4: age, sex, income quartile, BMI, smoking status, drinking habit, physical activity, hypertension, dyslipidemia, chronic kidney disease, heart failure, history of myocardial infarction, and history of ischemic stroke; Model 5: Model 3 + diabetic retinopathy and diabetic neuropathy; Model 6: Model 4 + diabetic retinopathy and diabetic neuropathy

AF: atrial fibrillation; PY: person-years; 95% CI: 95% confidence interval; ref.: reference value for hazard ratio; Neg: negative urine dipstick test; DM: diabetes mellitus; C-index: Harrell's C-index

Fig S1. Kaplan-Meier curves for AF incidence corresponding proteinuria level for each glycemic stage.

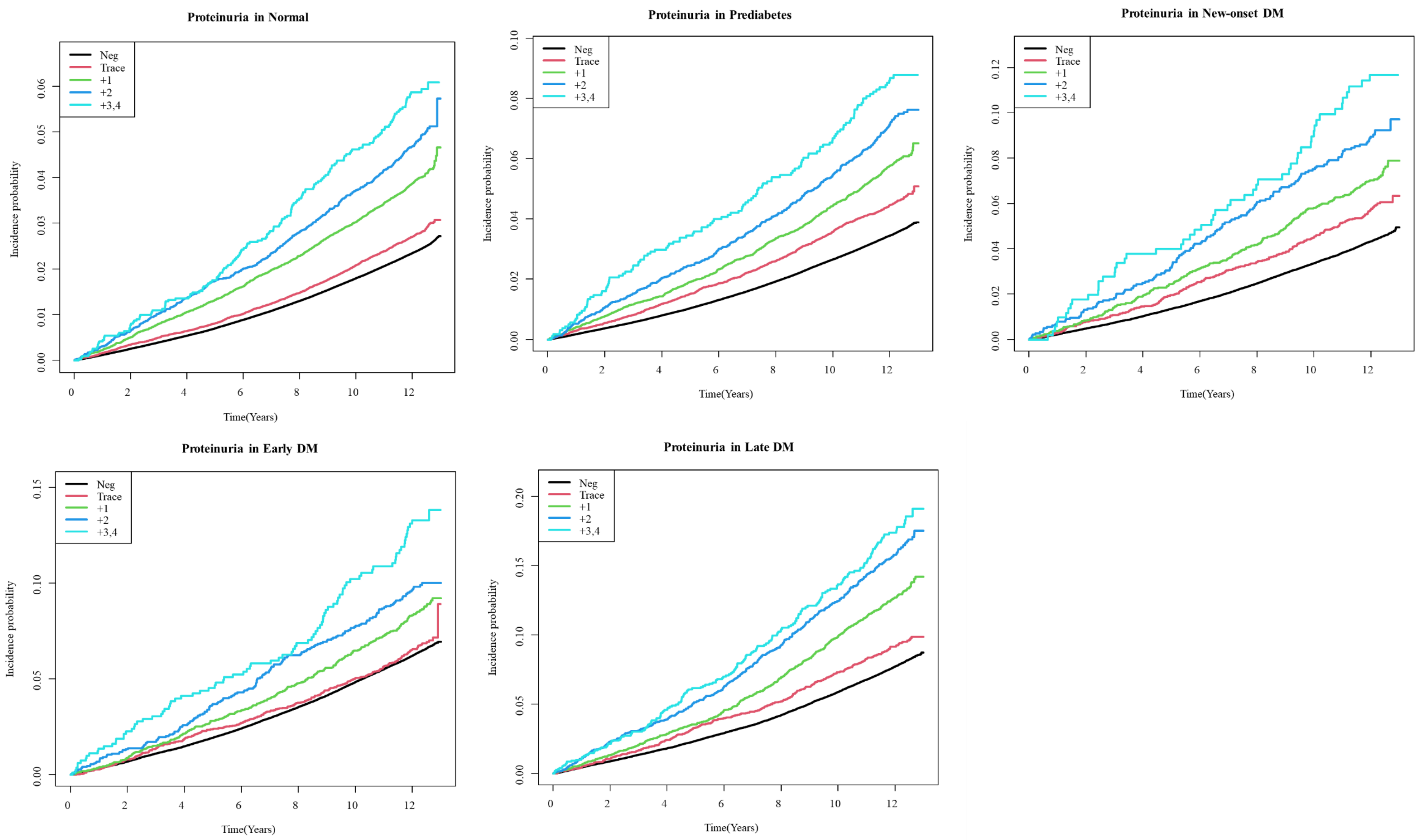

Supplement: Supplementary file 1 — Additional file 1. Table S1. Definitions of variables in the study. Table S2. Hazard ratios of AF incidence rate by proteinuria level within each glycemic stage (negative proteinuria in each glycemic stage as reference). Figure S1. Kaplan-Meier curves for AF incidence corresponding proteinuria level for each glycemic stage. [file 12933_2025_2590_MOESM1_ESM.pdf]
