# Supplementary material for: Proteinuria and the risk of Incident atrial fibrillation according to glycemic stages: a nationwide population-based cohort study
Source: Cardiovasc Diabetol. 2025 Jan 24;24:41. doi: 10.1186/s12933-025-02590-2 (PMC11762047; doi:10.1186/s12933-025-02590-2)

**Table S3. Incidence rates of AF across glycemic stages and proteinuria levels using competing risk models.**

| Groups | N | Event | Deaths | Duration* | AF IR* | Sub-distribution Hazard Ratio (95% CI) | | | | | |
|---|---|---|---|---|---|---|---|---|---|---|---|
| | | | | | | Model 1 | Model 2 | Model 3 | Model 4 | Model 5 | Model 6 |
| Glycemic Stages | | | | | | W | | | | | |
| Normal | 2,783,623 | 68,388 | 124,852 | 33394.4 | 2.05 | 1 (ref.) | 1 (ref.) | 1 (ref.) | 1 (ref.) | 1 (ref.) | 1 (ref.) |
| Prediabetes | 915,914 | 32,892 | 61,585 | 10818.8 | 3.04 | 1.47 (1.45, 1.49) | 1.09 (1.08, 1.11) | 1.01 (0.99, 1.02) | 1.01 (1.00, 1.02) | 1.01 (0.99, 1.02) | 1.01 (1.00, 1.02) |
| New-onset DM | 120,758 | 5,423 | 13,219 | 1387.3 | 3.91 | 1.85 (1.80, 1.91) | 1.19 (1.16, 1.22) | 1.04 (1.01, 1.07) | 1.04 (1.02, 1.08) | 1.04 (1.01, 1.07) | 1.04 (1.01, 1.07) |
| Early DM | 119,710 | 7,417 | 19,107 | 1342.0 | 5.53 | 2.56 (2.50, 2.62) | 1.21 (1.18, 1.24) | 1.01 (0.98, 1.03) | 1.00 (0.97, 1.02) | 0.98 (0.96, 1.01) | 0.98 (0.95, 1.00) |
| Late DM | 104519 | 8104 | 24175 | 1121.8 | 7.22 | 3.24 (3.17, 3.32) | 1.27 (1.25, 1.30) | 1.08 (1.06, 1.11) | 1.07 (1.04, 1.09) | 1.03 (1.00, 1.06) | 1.02 (0.99, 1.05) |
| p-value | | | | | | <.0001 | <.0001 | <.0001 | 0.0031 | <.0001 | 0.0023 |
| Proteinuria Levels | | | | | | | | | | | |
| Negative | 3,852,974 | 112,808 | 222,537 | 45863.5 | 2.46 | 1 (Ref.) | 1 (Ref.) | 1 (Ref.) | 1 (Ref.) | | |
| Trace | 91,593 | 3,421 | 7,277 | 1074.1 | 3.18 | 1.28 (1.24, 1.33) | 1.12 (1.08, 1.16) | 1.07 (1.03, 1.10) | 1.06 (1.03, 1.10) | | |
| 1 | 67,532 | 3,618 | 7,552 | 772.4 | 4.68 | 1.86 (1.80, 1.92) | 1.41 (1.36, 1.46) | 1.28 (1.24, 1.33) | 1.28 (1.24, 1.32) | | |
| 2 | 24,877 | 1,726 | 3,938 | 274.6 | 6.29 | 2.43 (2.31, 2.55) | 1.67 (1.59, 1.75) | 1.46 (1.40, 1.54) | 1.45 (1.38, 1.52) | | |
| +3,4 | 7,548 | 651 | 1,634 | 79.6 | 8.18 | 3.06 (2.83, 3.30) | 1.91 (1.76, 2.07) | 1.64 (1.51, 1.77) | 1.62 (1.49, 1.75) | | |
| p-value | | | | | | <.0001 | <.0001 | <.0001 | <.0001 | | |

Model 1: non-adjusted; Model 2: age, sex; Model 3: age, sex, income quartile, body-mass index (BMI), smoking status, drinking habit, physical activity, hypertension, dyslipidemia, and chronic kidney disease; Model 4: age, sex, income quartile, BMI, smoking status, drinking habit, physical activity, hypertension, dyslipidemia, chronic kidney disease, heart failure, history of myocardial infarction, and history of ischemic stroke; Model 5: Model 3 + diabetic retinopathy and diabetic neuropathy; Model 6: Model 4 + diabetic retinopathy and diabetic neuropathy.

AF: atrial fibrillation; IR: incidence rate; 95% CI: 95% confidence interval; ref.: reference value of sub-distribution hazard ratio; DM: diabetes mellitus; Neg: negative urine dipstick test.

* Per 1000 person-years

**Table S4. Hazard ratios of AF incidence rate by proteinuria level within each glycemic stage using competing risk models.**

| Glycemic stages | Protein uria | N | Events | Deaths | Duration* | AF IR* | Sub-Distribution Hazard Ratio (95% CI) | | | | | |
|---|---|---|---|---|---|---|---|---|---|---|---|---|
| | | | | | | | Model 1 | Model 2 | Model 3 | Model 4 | Model 5 | Model 6 |
| Normal | Neg | 2,674,628 | 64,623 | 117,732 | 32,107.6 | 2.01 | 1 (ref.) | 1 (ref.) | 1 (ref.) | 1 (ref.) | 1 (ref.) | 1 (ref.) |
| | Trace | 56,703 | 1,573 | 2,998 | 676.0 | 2.33 | 1.15 (1.09, 1.21) | 1.08 (1.03, 1.14) | 1.04 (0.99, 1.10) | 1.04 (0.99, 1.10) | 1.04 (0.99, 1.10) | 1.04 (0.99, 1.10) |
| | +1 | 37,062 | 1,449 | 2,627 | 435.3 | 3.33 | 1.63 (1.55, 1.72) | 1.35 (1.28, 1.42) | 1.25 (1.19, 1.32) | 1.25 (1.19, 1.32) | 1.25 (1.19, 1.32) | 1.25 (1.19, 1.32) |
| | +2 | 12,089 | 568 | 1,117 | 139.9 | 4.06 | 1.97 (1.81, 2.14) | 1.52 (1.40, 1.66) | 1.36 (1.25, 1.48) | 1.35 (1.24, 1.47) | 1.36 (1.25, 1.48) | 1.35 (1.24, 1.47) |
| | +3,4 | 3,141 | 175 | 378 | 35.6 | 4.91 | 2.35 (2.03, 2.73) | 1.65 (1.42, 1.92) | 1.46 (1.25, 1.69) | 1.45 (1.24, 1.68) | 1.45 (1.25, 1.69) | 1.44 (1.24, 1.68) |
| Prediabetes | Neg | 870,724 | 30,492 | 57,016 | 10,298.7 | 2.96 | 1.46 (1.44, 1.48) | 1.09 (1.07, 1.10) | 1.01 (0.99, 1.02) | 1.01 (0.99, 1.02) | 1.01 (0.99, 1.02) | 1.01 (0.99, 1.02) |
| | Trace | 22,000 | 980 | 1,897 | 256.4 | 3.82 | 1.87 (1.75, 1.99) | 1.22 (1.15, 1.30) | 1.08 (1.02, 1.15) | 1.08 (1.01, 1.15) | 1.08 (1.02, 1.15) | 1.08 (1.01, 1.15) |
| | +1 | 16,138 | 914 | 1,690 | 185.0 | 4.94 | 2.39 (2.24, 2.55) | 1.45 (1.35, 1.55) | 1.24 (1.16, 1.32) | 1.24 (1.16, 1.33) | 1.24 (1.16, 1.32) | 1.24 (1.16, 1.33) |
| | +2 | 5,483 | 379 | 741 | 61.4 | 6.17 | 2.93 (2.65, 3.24) | 1.63 (1.47, 1.81) | 1.36 (1.23, 1.51) | 1.36 (1.23, 1.50) | 1.36 (1.23, 1.51) | 1.36 (1.22, 1.50) |
| | +3,4 | 1,569 | 127 | 241 | 17.2 | 7.38 | 3.48 (2.92, 4.14) | 1.79 (1.49, 2.14) | 1.47 (1.23, 1.76) | 1.46 (1.22, 1.76) | 1.46 (1.22, 1.76) | 1.46 (1.22, 1.75) |
| New-onset DM | Neg | 110,163 | 4,708 | 11,630 | 1,269.9 | 3.71 | 1.79 (1.74, 1.85) | 1.15 (1.12, 1.19) | 1.02 (0.99, 1.05) | 1.02 (0.99, 1.05) | 1.01 (0.98, 1.04) | 1.02 (0.99, 1.05) |
| | Trace | 4,156 | 233 | 537 | 47.0 | 4.95 | 2.37 (2.08, 2.70) | 1.43 (1.25, 1.63) | 1.22 (1.07, 1.39) | 1.22 (1.07, 1.39) | 1.21 (1.06, 1.38) | 1.22 (1.07, 1.39) |
| | +1 | 4,159 | 282 | 609 | 46.2 | 6.11 | 2.89 (2.57, 3.25) | 1.70 (1.51, 1.92) | 1.41 (1.26, 1.59) | 1.41 (1.25, 1.59) | 1.41 (1.25, 1.58) | 1.41 (1.25, 1.58) |
| | +2 | 1,765 | 147 | 324 | 18.8 | 7.80 | 3.59 (3.05, 4.23) | 2.04 (1.72, 2.41) | 1.67 (1.41, 1.98) | 1.67 (1.42, 1.98) | 1.66 (1.40, 1.96) | 1.66 (1.41, 1.97) |
| | +3,4 | 515 | 53 | 119 | 5.3 | 9.93 | 4.50 (3.43, 5.90) | 2.49 (1.88, 3.29) | 2.01 (1.52, 2.65) | 2.00 (1.51, 2.64) | 1.98 (1.50, 2.62) | 1.98 (1.50, 2.61) |
| Early DM | Neg | 107,415 | 6,479 | 16,607 | 1,209.1 | 5.36 | 2.53 (2.47, 2.60) | 1.19 (1.16, 1.22) | 1.00 (0.97, 1.02) | 0.99 (0.96, 1.01) | 0.98 (0.95, 1.00) | 0.97 (0.94, 1.00) |
| | Trace | 4,540 | 285 | 793 | 50.5 | 5.65 | 2.64 (2.35, 2.97) | 1.24 (1.10, 1.40) | 1.00 (0.89, 1.13) | 1.00 (0.88, 1.12) | 0.98 (0.87, 1.11) | 0.98 (0.87, 1.10) |
| | +1 | 4,746 | 371 | 954 | 51.4 | 7.22 | 3.32 (3.00, 3.68) | 1.55 (1.40, 1.72) | 1.23 (1.10, 1.36) | 1.22 (1.10, 1.35) | 1.20 (1.08, 1.33) | 1.19 (1.08, 1.32) |
| | +2 | 2,198 | 191 | 519 | 22.9 | 8.32 | 3.73 (3.24, 4.30) | 1.80 (1.56, 2.09) | 1.39 (1.20, 1.61) | 1.38 (1.19, 1.59) | 1.35 (1.17, 1.56) | 1.34 (1.16, 1.55) |
| | +3,4 | 811 | 91 | 234 | 8.1 | 11.27 | 4.88 (3.97, 6.00) | 2.33 (1.88, 2.88) | 1.77 (1.43, 2.19) | 1.75 (1.41, 2.16) | 1.72 (1.39, 2.12) | 1.70 (1.38, 2.10) |
| Late DM | Neg | 90,044 | 6,506 | 19,552 | 978.2 | 6.65 | 3.06 (2.98, 3.14) | 1.20 (1.17, 1.24) | 1.03 (1.01, 1.06) | 1.02 (0.99, 1.04) | 0.99 (0.96, 1.02) | 0.98 (0.95, 1.01) |
| | Trace | 4,194 | 350 | 1,052 | 44.2 | 7.93 | 3.58 (3.22, 3.97) | 1.37 (1.23, 1.52) | 1.14 (1.02, 1.26) | 1.11 (1.00, 1.24) | 1.08 (0.97, 1.20) | 1.07 (0.96, 1.19) |
| | +1 | 5,427 | 602 | 1,672 | 54.6 | 11.03 | 4.81 (4.44, 5.22) | 1.81 (1.67, 1.97) | 1.48 (1.36, 1.60) | 1.45 (1.33, 1.57) | 1.40 (1.29, 1.52) | 1.39 (1.28, 1.51) |
| | +2 | 3,342 | 441 | 1,237 | 31.5 | 14.00 | 5.85 (5.32, 6.42) | 2.19 (1.99, 2.42) | 1.76 (1.60, 1.94) | 1.70 (1.54, 1.87) | 1.66 (1.51, 1.83) | 1.62 (1.46, 1.79) |
| | +3,4 | 1,512 | 205 | 662 | 13.4 | 15.33 | 6.06 (5.28, 6.96) | 2.37 (2.06, 2.73) | 1.86 (1.61, 2.14) | 1.79 (1.55, 2.06) | 1.74 (1.50, 2.00) | 1.69 (1.46, 1.95) |
| p-value | | | | | | | <.0001 | <.0001 | <.0001 | <.0001 | <.0001 | <.0001 |

| Glycemic stages | Protein uria | N | Events | Deaths | Duration* | AF IR* | Sub-Distribution Hazard ratio (95% CI) | | | | | |
|---|---|---|---|---|---|---|---|---|---|---|---|---|
| | | | | | | | Model 1 | Model 2 | Model 3 | Model 4 | Model 5 | Model 6 |
| Normal | Neg | 2,674,628 | 64,623 | 117,732 | 32,107.6 | 2.01 | 1 (ref.) | 1 (ref.) | 1 (ref.) | 1 (ref.) | 1 (ref.) | 1 (ref.) |
| | Trace | 56,703 | 1,573 | 2,998 | 676.0 | 2.33 | 1.15 (1.09, 1.21) | 1.08 (1.03, 1.14) | 1.04 (0.99, 1.10) | 1.04 (0.99, 1.10) | 1.04 (0.99, 1.10) | 1.04 (0.99, 1.10) |
| | +1 | 37,062 | 1,449 | 2,627 | 435.3 | 3.33 | 1.63 (1.55, 1.72) | 1.35 (1.28, 1.42) | 1.25 (1.19, 1.32) | 1.25 (1.19, 1.32) | 1.25 (1.19, 1.32) | 1.25 (1.19, 1.32) |
| | +2 | 12,089 | 568 | 1,117 | 139.9 | 4.06 | 1.97 (1.81, 2.14) | 1.52 (1.40, 1.66) | 1.36 (1.25, 1.48) | 1.35 (1.24, 1.47) | 1.36 (1.25, 1.48) | 1.35 (1.24, 1.47) |
| | +3,4 | 3,141 | 175 | 378 | 35.6 | 4.91 | 2.35 (2.03, 2.73) | 1.65 (1.42, 1.92) | 1.46 (1.25, 1.69) | 1.45 (1.24, 1.68) | 1.45 (1.25, 1.69) | 1.44 (1.24, 1.68) |
| Prediabetes | Neg | 870,724 | 30,492 | 57,016 | 10,298.7 | 2.96 | 1 (ref.) | 1 (ref.) | 1 (ref.) | 1 (ref.) | 1 (ref.) | 1 (ref.) |
| | Trace | 22,000 | 980 | 1,897 | 256.4 | 3.82 | 1.28 (1.20, 1.36) | 1.12 (1.05, 1.20) | 1.08 (1.01, 1.15) | 1.07 (1.01, 1.14) | 1.08 (1.01, 1.15) | 1.07 (1.01, 1.14) |
| | +1 | 16,138 | 914 | 1,690 | 185.0 | 4.94 | 1.64 (1.53, 1.75) | 1.33 (1.24, 1.42) | 1.23 (1.15, 1.32) | 1.23 (1.15, 1.32) | 1.23 (1.15, 1.32) | 1.23 (1.15, 1.32) |
| | +2 | 5,483 | 379 | 741 | 61.4 | 6.17 | 2.01 (1.82, 2.22) | 1.50 (1.35, 1.66) | 1.36 (1.22, 1.50) | 1.35 (1.22, 1.49) | 1.35 (1.22, 1.50) | 1.35 (1.21, 1.49) |

| | | | | | | | | | | | | |
|---|---|---|---|---|---|---|---|---|---|---|---|---|
| | +3,4 | 1,569 | 127 | 241 | 17.2 | 7.38 | 2.38 (2.00, 2.84) | 1.64 (1.37, 1.97) | 1.46 (1.22, 1.75) | 1.45 (1.21, 1.74) | 1.46 (1.22, 1.74) | 1.45 (1.21, 1.74) |
| New-onset DM | Neg | 110,163 | 4,708 | 11,630 | 1,269.9 | 3.71 | 1 (ref.) | 1 (ref.) | 1 (ref.) | 1 (ref.) | 1 (ref.) | 1 (ref.) |
| | Trace | 4,156 | 233 | 537 | 47.0 | 4.95 | 1.32 (1.16, 1.51) | 1.24 (1.09, 1.42) | 1.20 (1.05, 1.37) | 1.20 (1.05, 1.37) | 1.20 (1.05, 1.37) | 1.20 (1.05, 1.37) |
| | +1 | 4,159 | 282 | 609 | 46.2 | 6.11 | 1.61 (1.43, 1.82) | 1.48 (1.31, 1.67) | 1.39 (1.23, 1.57) | 1.39 (1.23, 1.57) | 1.39 (1.23, 1.57) | 1.38 (1.23, 1.56) |
| | +2 | 1,765 | 147 | 324 | 18.8 | 7.80 | 2.00 (1.70, 2.37) | 1.77 (1.49, 2.10) | 1.64 (1.39, 1.95) | 1.64 (1.39, 1.95) | 1.64 (1.38, 1.94) | 1.64 (1.38, 1.94) |
| | +3,4 | 515 | 53 | 119 | 5.3 | 9.93 | 2.51 (1.91, 3.30) | 2.16 (1.63, 2.86) | 1.98 (1.50, 2.61) | 1.96 (1.48, 2.59) | 1.96 (1.48, 2.58) | 1.95 (1.47, 2.57) |
| Early DM | Neg | 107,415 | 6,479 | 16,607 | 1,209.1 | 5.36 | 1 (ref.) | 1 (ref.) | 1 (ref.) | 1 (ref.) | 1 (ref.) | 1 (ref.) |
| | Trace | 4,540 | 285 | 793 | 50.5 | 5.65 | 1.04 (0.93, 1.17) | 1.04 (0.92, 1.18) | 1.01 (0.89, 1.14) | 1.01 (0.89, 1.14) | 1.01 (0.89, 1.14) | 1.01 (0.89, 1.14) |
| | +1 | 4,746 | 371 | 954 | 51.4 | 7.22 | 1.31 (1.18, 1.46) | 1.30 (1.17, 1.44) | 1.23 (1.11, 1.37) | 1.23 (1.11, 1.37) | 1.23 (1.10, 1.36) | 1.23 (1.11, 1.37) |
| | +2 | 2,198 | 191 | 519 | 22.9 | 8.32 | 1.47 (1.27, 1.70) | 1.52 (1.31, 1.76) | 1.40 (1.20, 1.62) | 1.39 (1.20, 1.62) | 1.38 (1.20, 1.60) | 1.38 (1.19, 1.60) |
| | +3,4 | 811 | 91 | 234 | 8.1 | 11.27 | 1.93 (1.56, 2.37) | 1.96 (1.58, 2.42) | 1.78 (1.44, 2.20) | 1.77 (1.43, 2.19) | 1.76 (1.42, 2.18) | 1.75 (1.42, 2.17) |
| Late DM | Neg | 90,044 | 6,506 | 19,552 | 978.2 | 6.65 | 1 (ref.) | 1 (ref.) | 1 (ref.) | 1 (ref.) | 1 (ref.) | 1 (ref.) |
| | Trace | 4,194 | 350 | 1,052 | 44.2 | 7.93 | 1.17 (1.05, 1.30) | 1.14 (1.02, 1.27) | 1.10 (0.98, 1.23) | 1.09 (0.98, 1.22) | 1.09 (0.98, 1.22) | 1.09 (0.98, 1.22) |
| | +1 | 5,427 | 602 | 1,672 | 54.6 | 11.03 | 1.57 (1.45, 1.71) | 1.51 (1.38, 1.64) | 1.43 (1.31, 1.56) | 1.42 (1.30, 1.55) | 1.42 (1.30, 1.54) | 1.41 (1.30, 1.54) |
| | +2 | 3,342 | 441 | 1,237 | 31.5 | 14.00 | 1.91 (1.73, 2.10) | 1.82 (1.65, 2.01) | 1.70 (1.54, 1.88) | 1.66 (1.51, 1.84) | 1.68 (1.52, 1.86) | 1.65 (1.49, 1.82) |
| | +3,4 | 1,512 | 205 | 662 | 13.4 | 15.33 | 1.98 (1.72, 2.28) | 1.97 (1.70, 2.27) | 1.80 (1.56, 2.07) | 1.76 (1.52, 2.03) | 1.76 (1.52, 2.03) | 1.72 (1.49, 1.99) |
| p-value | | | | | | | <.0001 | 0.0304 | 0.0027 | 0.0061 | 0.0065 | 0.0123 |

Model 1: non-adjusted; Model 2: age, sex; Model 3: age, sex, income quartile, body-mass index (BMI), smoking status, drinking habit, physical activity, hypertension, dyslipidemia, and chronic kidney disease; Model 4: age, sex, income quartile, BMI, smoking status, drinking habit, physical activity, hypertension, dyslipidemia, chronic kidney disease, heart failure, history of myocardial infarction, and history of ischemic stroke; Model 5: Model 3 + diabetic retinopathy and diabetic neuropathy; Model 6: Model 4 + diabetic retinopathy and diabetic neuropathy.

AF: atrial fibrillation; IR: incidence rate; 95% CI: 95% confidence interval; ref.: reference value of sub-distribution hazard ratio; DM: diabetes mellitus; Neg: negative urine dipstick test.

[*] Per 1000 person-years

**Fig S2. Cumulative incidence curves for AF incidence corresponding to glycemic stages and proteinuria level using competing risk models.**

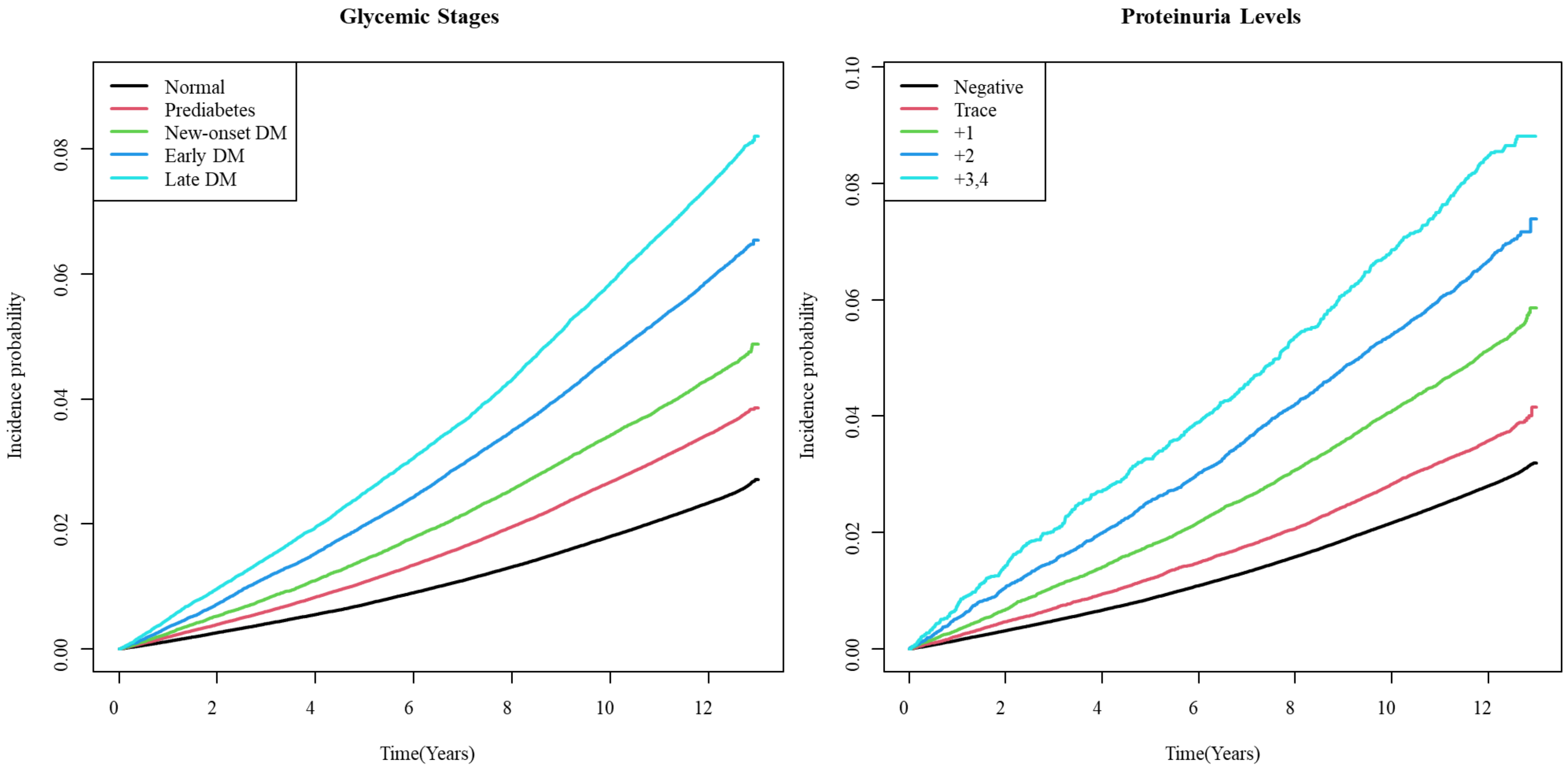

**Fig S3. Cumulative incidence curves for AF incidence corresponding proteinuria level for each glycemic stage using competing risk models.**

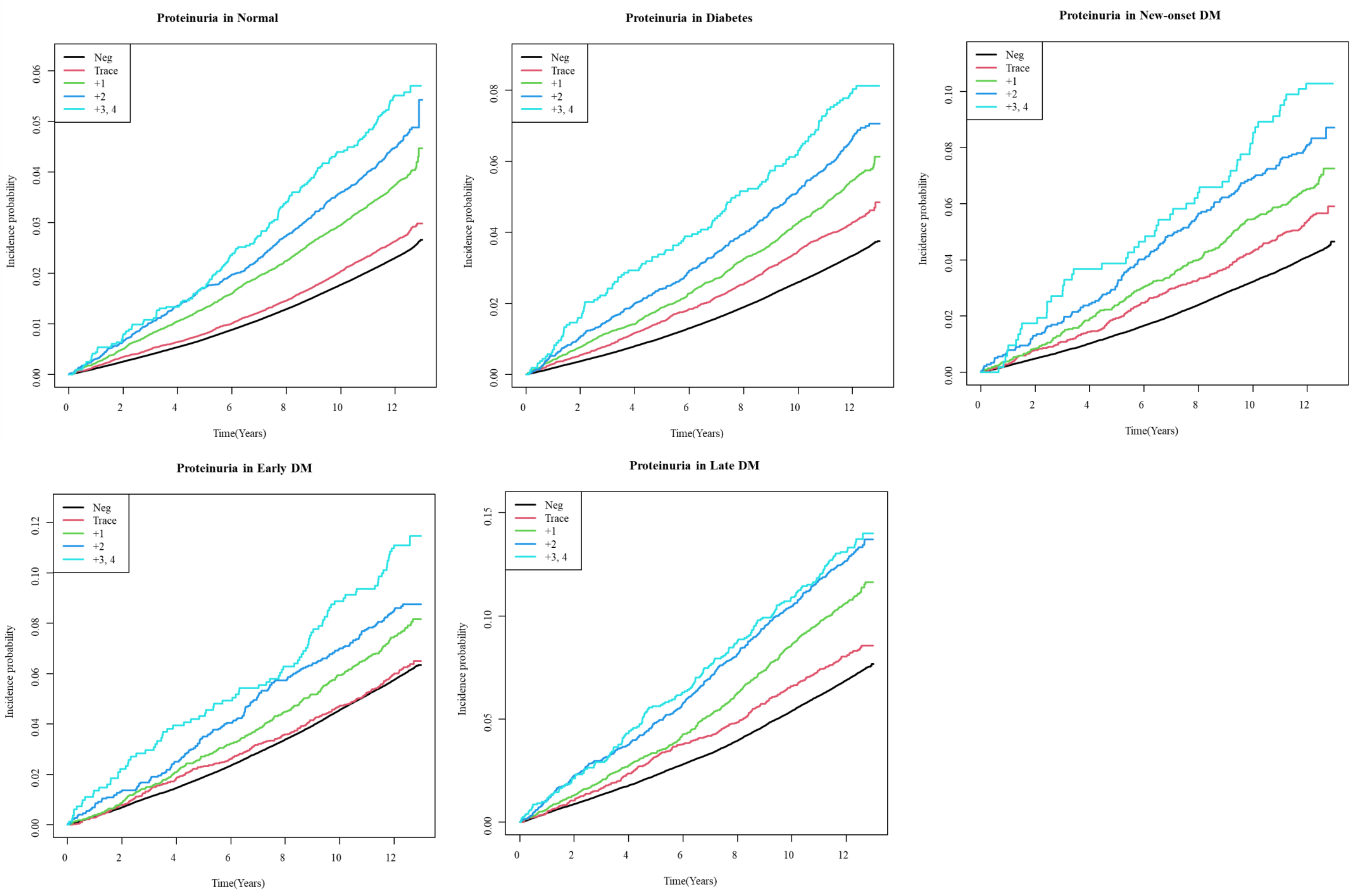

**Fig S4. AF incidence rates and hazard ratios in Cox Proportional-Hazards Regression Model 4 with competing risks.**

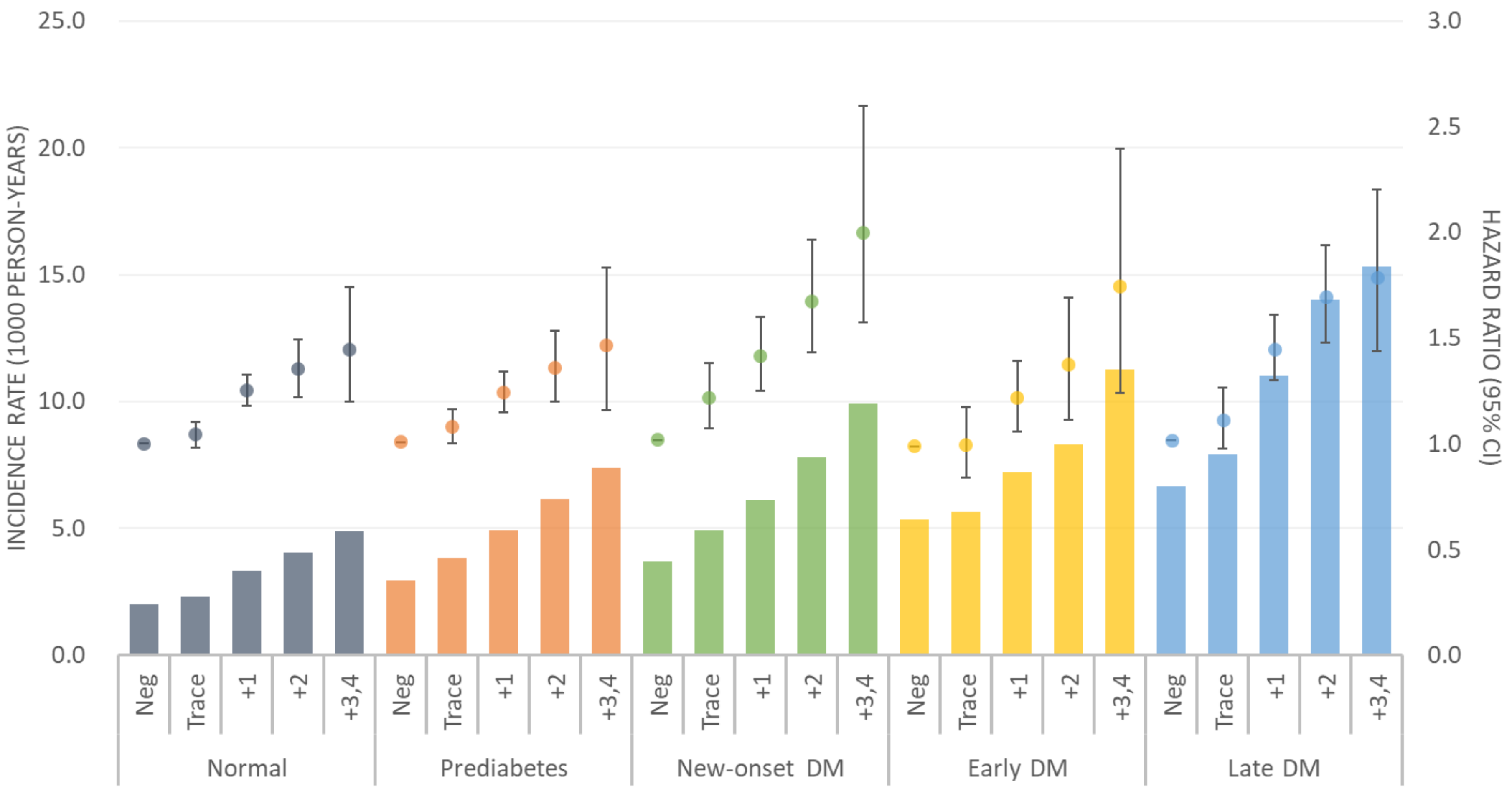

Supplement: Supplementary file 2 — Additional file 2. Table S3. Incidence rates of AF across glycemic stages and proteinuria levels using competing risk models. Table S4. Hazard ratios of AF incidence rate by proteinuria level within each glycemic stage using competing risk models. Figure S2. Cumulative incidence curves for AF incidence corresponding to glycemic stages and proteinuria level using competing risk models.Figure S3. Cumulative incidence curves for AF incidence corresponding proteinuria level for each glycemic stage using competing risk models. Figure S4. AF incidence rates and hazard ratios in Cox Proportional-Hazards Regression Model 4 with competing risks. [file 12933_2025_2590_MOESM2_ESM.pdf]
